# The DLGAP family: neuronal expression, function and role in brain disorders

**DOI:** 10.1186/s13041-017-0324-9

**Published:** 2017-09-04

**Authors:** Andreas H. Rasmussen, Hanne B. Rasmussen, Asli Silahtaroglu

**Affiliations:** 1 Department of Cellular and Molecular Medicine, Faculty of Medical and Health Sciences, University of Copenhagen, DK-2200 Copenhagen, Denmark; 2 Department of Biomedical Sciences, Faculty of Medical and Health Sciences, University of Copenhagen, DK-2200 Copenhagen, Denmark

**Keywords:** DLGAP1, DLGAP2, DLGAP3, DLGAP4, SAPAP, PSD, GKAP, Schizophrenia, Scaffold proteins, Synaptic scaling

## Abstract

The neurotransmitter glutamate facilitates neuronal signalling at excitatory synapses. Glutamate is released from the presynaptic membrane into the synaptic cleft. Across the synaptic cleft glutamate binds to both ion channels and metabotropic glutamate receptors at the postsynapse, which expedite downstream signalling in the neuron. The postsynaptic density, a highly specialized matrix, which is attached to the postsynaptic membrane, controls this downstream signalling. The postsynaptic density also resets the synapse after each synaptic firing. It is composed of numerous proteins including a family of Discs large associated protein 1, 2, 3 and 4 (DLGAP1-4) that act as scaffold proteins in the postsynaptic density. They link the glutamate receptors in the postsynaptic membrane to other glutamate receptors, to signalling proteins and to components of the cytoskeleton. With the central localisation in the postsynapse, the DLGAP family seems to play a vital role in synaptic scaling by regulating the turnover of both ionotropic and metabotropic glutamate receptors in response to synaptic activity. DLGAP family has been directly linked to a variety of psychological and neurological disorders. In this review we focus on the direct and indirect role of DLGAP family on schizophrenia as well as other brain diseases.

## Introduction

The postsynaptic density (PSD) is a highly specialized matrix that is involved in transmission of neuronal signals across the synaptic junction. The PSD is found in the synaptic terminal of postsynaptic neurons. A variety of proteins is expressed in the PSD for transmitting neuronal signals from the PSD to the soma or other parts of the neuron. The PSD scaffolding proteins, Discs large scaffold proteins (DLGs) and the family of Src-homology (SH3) and multiple ankyrin repeat domain proteins (SHANK) are essential for proper function of both the ionotropic N-methyl-D-aspartate (NMDA) and α-amino-3-hydroxy-5-methyl-4-isoxazolepropionic acid (AMPA) receptors and the metabotropic glutamate receptors (mGluRs) [1–4]. The family of Discs large associated proteins (DLGAPs) are also important scaffold proteins in the PSD. There are five DLGAP proteins, DLGAP1 – DLGAP5 where DLGAP1 - DLGAP4 (DLGAP1–4) proteins interact directly with both DLG and SHANK through their multiple domains [2, 5, 6]. Via interaction partners, DLGAP1–4 proteins are likely to play a role in multiple processes of the PSD. For example, neuronal DLGAP proteins have key roles in synaptic scaling [7, 8]. DLGAP1–4 proteins have also been linked to a variety of neurological disorders including schizophrenia, autism spectrum disease (ASD), trichotillomania, obsessive compulsive disorder (OCD) and cerebellar ataxia [9–14] (see Table 1). Here we present the existing knowledge on the expression, function and the regulation of DLGAP1–4 in the brain. We also review the link of DLGAP1–4 to the various neurological disorders. DLGAP5 is not included in this review since it is mainly linked to various types of cancers and does not seem to play an important role in neuronal signalling [15–17].

**Table 1:** Table with the DLGAP subtypes expressed in the brain i.e. DLGAP1–4 and the corresponding CNS diseases and affected brain regions

DLGAP subtype	CNS disease	Brain region affected	References
DLGAP1	Schizophrenia	Nucleus accumbens	[9]
	Alzheimer’s disease	Frontal cortex	[114]
	Major depressive disorder	Hippocampus	[115]
DLGAP2	Schizophrenia	na*	[99]
	Fragile x mental retardation	Hippocampus	[116]
	Post traumatic stress disorder	Hippocampus	[102]
	Autism spectrum disease	na*	[10, 100, 101]
DLGAP3	Trichotillomania	Striatum	[11, 12]
	Obsessive compulsive disorder	Striatum	[11, 13, 106]
	Parkinson’s disease	na*	[117]
	Schizophrenia	na*	[98]
DLGAP4	Cerebellar ataxia	Cerebellum	[14]
	Bipolar disorder (indirectly linked)	na*	[118]

na* No proven affected brain region. DLGAP1 is thought to be involved in schizophrenia starting from nucleus accumbens, Alzheimer’s disease in frontal cortex and major depressive disorder originating from hippocampus. DLGAP2 is linked to multiple diseases. First schizophrenia with no proven affected brain region, second autism spectrum disease also with no proven affected brain region and finally fragile x mental retardation and post-traumatic stress disorder both originating from the hippocampus. DLGAP3 is proven to be involved in both trichotillomania and OCD both starting in the striatum. DLGAP3 has also been linked to Parkinson’s disease. Finally, DLGAP4 is proven to be involved in cerebellar ataxia coming from cerebellum and indirectly to bipolar disorder via the microRNA miR-1908-5p. The relevant references are linked in the fourth column, respectively

### Nomenclature

The DLGAP protein family is known under several different names. Initially DLGAP1 was named Guanylate kinase associated protein (GKAP) since it interacts with the guanylate kinase (GK) domain of SAP90/PSD-95 [5]. Shortly after, three other similar proteins were and the family of four proteins was subsequently named as SAP90/PSD95-associated protein 1–4 (SAPAP1–4) [18]. DLGAP1 is, however, still referred to as GKAP in the literature. PSD-95 is also named Discs large scaffold protein 4 abbreviated DLG4. Therefore, the SAPAP family proteins were named discs large homolog associated protein (DLGAP) 1–4. PSD-95 will in this work be referred to as DLG4 and GKAP/SAPAP1–4 will be referred to as DLGAP1–4.

### *DLGAP* chromosomal localisation, protein homology and conservation

Four DLGAP proteins are found in the human brain, which are all encoded from a different locus. This section contains an overview of the chromosomal locations and mRNA transcript variants of *DLGAP* genes in human. The DLGAP proteins have a high sequential homology that we will review in detail along with the conservation of the DLGAP family in mammals and vertebrates.

#### Chromosomal localisation and mRNA transcripts

**Fig. 1:**
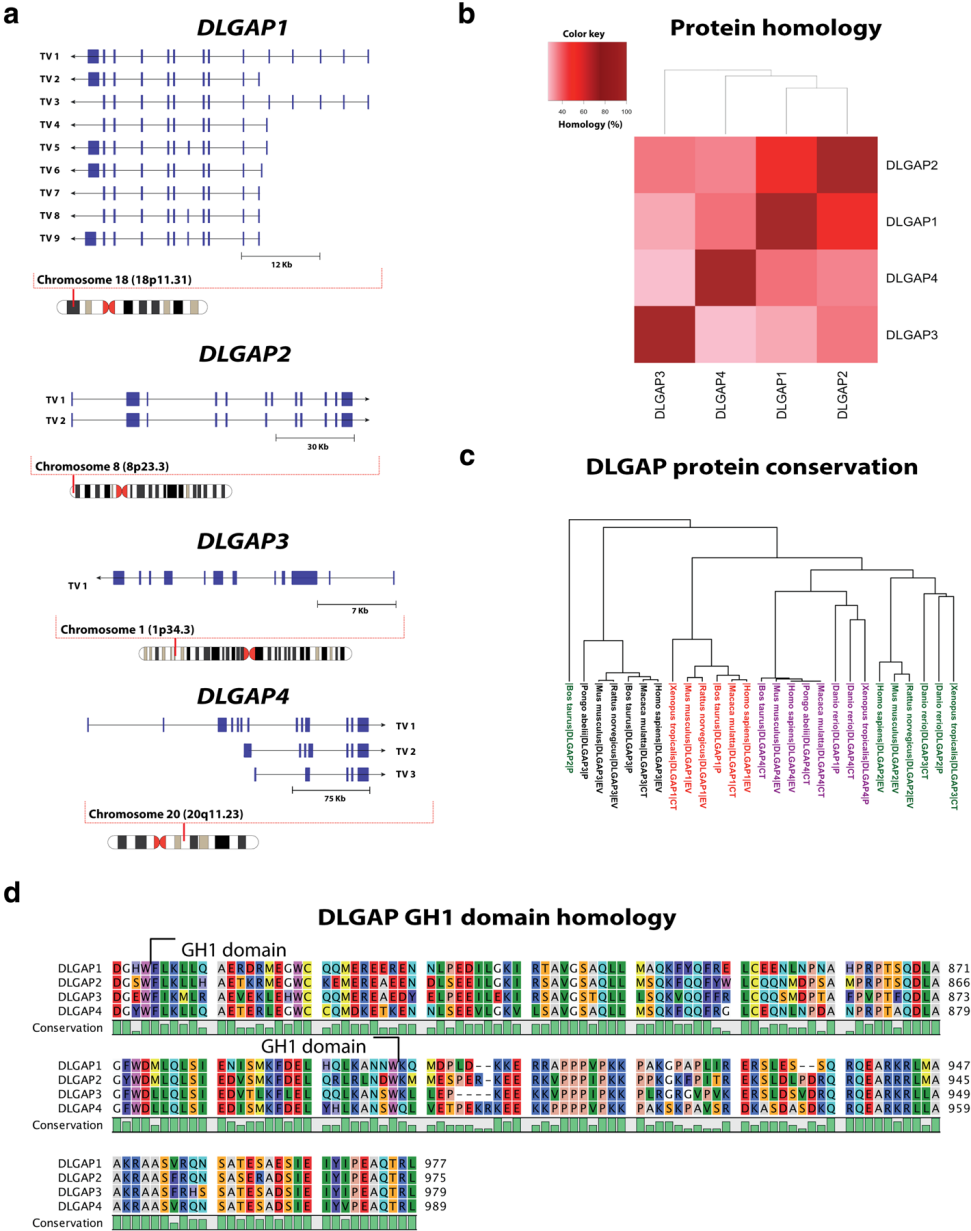
**a** Pictogram of chromosome 18, 8, 1 and 20 and the four DLGAP1–4 loci, respectively. DLGAP1 is located on chromosome 18 and transcript variants 1 to 9 are illustrated. DLGAP2 is found on chromosome 8 and has two transcript variants. The DLGAP3 gene is located on chromosome 1 and has one transcript variant. DLGAP4 is located on chromosome 20 and has 3 transcript variants. **b** Heatmap showing the homology in percentage between the longest isoform, isoform a, of DLGAP1–4 proteins respectively. The color key indicates the respective percentage to each colour. The heatmap is created in RStudio v.0.99.484 with data from a multiple alignment made with clustal omega v.1.2.1. DLGAP1 isoform a: 977 amino acids (NP_004737.2), DLGAP2 isoform a: 975 amino acids (NP_004736.2), DLGAP3 isoform a: 979 amino acids (NP_001073887.1) and DLGAP4 isoform a: 989 amino acids (NP_055717.2). **c** Phylogenetic tree of DLGAP1–4 protein conservation in following species; *Homo sapiens* (NP_004737.2, NP_004736.2, NP_001073887.1, NP_001035951.1), *Macaca mulatta* (AFE64413.1, AFJ72104.1, AFE64177.1), *Bos taurus* (NP_001179558.1, DAA17363.1, NP_001179367.1, AAI26739.1), *Rattus norvegicus* (NP_075235.3, NP_446353.2, NP_775161.2), *Mus musculus* (NP_808307.2, NP_766498.2, AAH57615.1, NP_001035953.1), *Pongo abelii* (XP_009251088.1, NP_001127321.1), *Xenopus tropicalis* (NP_001123829.1, NP_001106458.1, XP_012827022.1) and *Danio rerio* (NP_001189384.1, XP_009291347.1, NP_001038179.1, AAI33919.1). EV: Experimentally validated, CT: Conceptual Translation, P: Predicted from genomic sequence. The phylogenetic tree is created in RStudio v.0.99.484 with data from a multiple alignment made with clustal omega v.1.2.1. **d** Figure of multiple alignment of DLGAP1–4 GH1 domain and C-terminal. The sequence homology of the GH1 domain is 61 to 75% between DLGAP1 – DLGAP4. The alignment and data is generated in clustal omega v.1.2.1 and visualized in CLC sequence viewer v.7.6

The *DLGAP1–4* genes are located on 4 different chromosomes. *DLGAP1* is located on the short arm of chromosome 18 within the band 11.31 (18p11.31) and has 9 human transcript variants spanning from 2253 nucleotides to 6628 nucleotides (see Fig. 1a). *DLGAP2* is located on the short arm of chromosome 8, in the band 23.3 (8p23.3). Two transcript variants have been identified for *DLGAP2* having only one exon difference. Transcript variant 1 has 12 exons and transcript variant 2 has 11 exons (see Fig. 1a). *DLGAP3* is located on chromosome 1 in the band 34.3 on the short arm (1p34.3). Only one human *DLGAP3* transcript has been identified so far (see Fig. 1a). The *DLGAP4* gene is situated on the long arm of chromosome 20 within the band 11.23 (20q11.23). Three transcript variants with varying lengths from 3044 nucleotides to 5080 nucleotides have been found in human (see Fig. 1a).

#### DLGAP protein homology

**Fig. 2:**
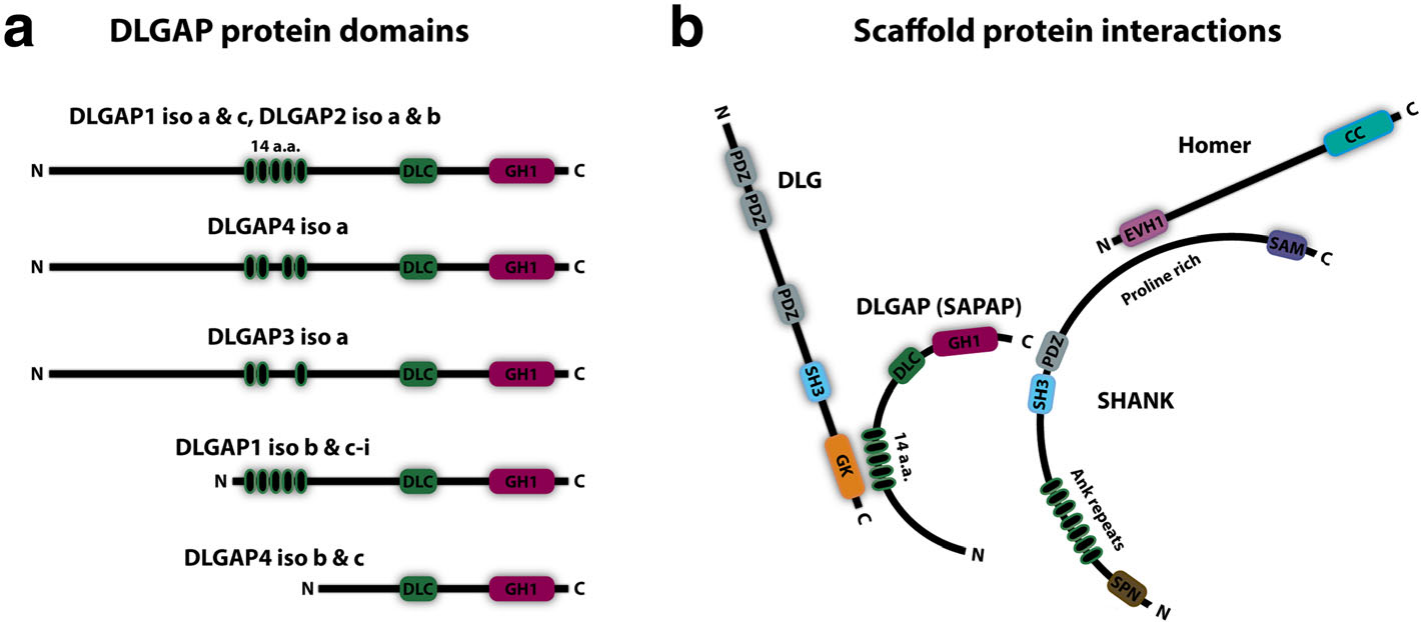
**a** Pictogram of the DLGAP1–4 proteins with their respective domains. The DLGAP1–4 proteins have a 14 amino acid repeat domain (14-a.a. repeats) with 0 to 5 repeats depending on the DLGAP isoform, a Dynein light chain (DLC) domain and a GKAP homology domain 1 (GH1). **b** Illustration of how the four proteins interact. DLGAP binds via the C-terminal to the PDZ domain of SHANK. The 14-a.a. repeat domain of DLGAP binds the GK domain of DLG. Homer binds with the EVH1 domain to the proline rich domain of SHANK

The many mRNA transcripts of *DLGAP1–4* encode DLGAP proteins with various lengths and domains (see Fig. 2a). The pairwise homology between the DLGAP proteins differ by 26 to 48%. DLGAP1 compared to DLGAP2 show the highest homology and DLGAP3 compared to DLGAP4 show the lowest homology (see Fig. 1b). DLGAP1–4 have 3 domains, a 14 amino acids repeat domain, a dynein light chain (DLC) domain and a GKAP homology (GH1) domain (see section 4.1). Especially the GH1 domain by which the DLGAP proteins are characterized, shows a high degree of homology i.e. 61 to 75%. In addition, the last 40 residues next to the C-terminal also show a high degree of homology (see Fig. 1d).

#### Conservation

DLGAP1–4 are important proteins in the postsynaptic density. They act as scaffold proteins and are involved in signalling to and from glutamate receptors [19]. The DLGAP proteins are conserved between species most likely because their function is highly essential. DLGAP proteins have been experimentally found or predicted from genomic sequences in *Homo sapiens, Macaca mulatta, Pongo abelii, Bos taurus, Rattus norvegicus, Mus musculus* and in the two vertebrates *Danio rerio* and *Xenopus tropicalis* (see Fig. 1c). DLGAP1–4 was originally found in rat [5, 18] but most research have since then been conducted in mouse and human where the genes and proteins also are described best.

### Domain overview and protein interaction

The DLGAP1–4 proteins contain several characterized domains that enable them to interact with numerous proteins in the PSD either directly or indirectly via other scaffold proteins (see Fig. 2a-b). Some of these interacting proteins, namely the DLG4 protein, the SHANK family, the Homer family and the Stargazin protein will also be reviewed here. These proteins all have domains that have the capability to link DLGAP1–4 to the three major glutamate receptors in the PSD, namely the NMDA receptor, the AMPA receptor and the group I mGluRs [1–3, 5, 20, 21].

#### The DLGAP family

As mentioned, the DLGAP proteins have three known domains, a 14-a.a. repeat domain, a DLC domain and a GH1 domain (see Fig. 2a). With the 14-a.a. domain DLGAP proteins are capable of binding the GK domain of DLG1, DLG2 and DLG4 [5, 22–24]. Depending on the isoform, the 14-a.a. domain can be located in the middle of the protein or near the N-terminal. For example, in the long DLGAP1 isoforms (isoform a & c) the repeats are located in the middle whereas in the short isoforms (isoform b, d-i) the repeats are located near the N-terminal. DLGAP1 has five 14-a.a. repeats but some of the repeats are missing in the other DLGAP proteins. For example, the long DLGAP4 isoform a lacks the repeat number 3 and the short isoforms b and c do not contain any of the 5 repeats (see Fig. 2a) [18]. This diversity in repeats may contribute to their individual function and binding affinity to DLG4 at the synapse. The second domain, the DLC domain is located downstream of the repeats. This DLC domain interacts directly with DLC, a motor protein subunit found in the dendrites and PSD [25]. The last domain, the GH1 domain, is found at the C-terminal. GH1 is composed of 4 alpha helices and display a high sequence homology between the DLGAP proteins as previously mentioned (see Figs. 1d and 2a & b). However, the role of the GH1 domain is still unknown [5, 6, 26].

#### Proteins interacting with the DLGAP family

##### The DLG family

The DLG family is a group of proteins also known as membrane-associated guanylate kinases (MAGUKs). Three of these proteins; DLG1, DLG2 and DLG4 also known as SAP97, PSD-93 and PSD-95, respectively, are known to interact directly with DLGAP [22–24]. The DLG proteins are important proteins in the PSD because they link other scaffold proteins and signalling molecules either directly or indirectly to the membrane bound ion channels. DLGs are known to stabilize the glutamate receptors upon binding and they may also trigger synaptic growth by modulation of growth-related proteins in the PSD [4]. DLGs, like DLGAP proteins, are also involved in synaptic scaling [4, 27–29], which is a process that is associated with schizophrenia [30]. The link of DLG1, DLG2 and DLG4 to schizophrenia has been well documented [31–36]. Additionally, DLG4 has been linked to ASD [37].

DLGs have three different types of domains where the first type is the Postsynaptic density protein; Drosophila disc large tumor suppressor; Zonula occludens-1 protein (PDZ) domain. In addition, DLGs have a Src Homology 3 (SH3) domain and a GK domain. DLGs have 3 PDZ domains (see Fig. 2b). The second PDZ domain binds the C-terminal of NMDA receptors [1]. Downstream of the PDZ domains, the SH3 domain is located, which has multiple binding partners including the A kinase anchor protein 150, AKAP150 [38]. Also, DLGs can create intramolecular interactions by binding the SH3 domain to the GK domain [39]. Most likely DLG proteins are also able to dimerize via SH3:GK interactions. With its GK domain DLGs interact directly with most DLGAP proteins through the 14-a.a. repeat domain [5] (see Fig. 2b). With the GK domain and the second PDZ domain DLGs acts as scaffolds between the DLGAP proteins and the NMDA receptors and AMPA receptors via Stargazin (see section 4.2.4) [3].

##### The SHANK family

DLGAP proteins bind a second family of proteins, the SHANK proteins [2]. Most SHANK proteins are highly expressed in the PSD and they are important for the maturation of the PSD. Overexpression of SHANK leads to earlier maturation of the postsynapse by recruitment and interaction with other scaffold proteins. There are three *SHANK* genes in humans that encode 3 SHANK proteins (SHANK1–3). *SHANK3* has been linked to schizophrenia whereas *SHANK1–3* have all been linked to ASD [40].

SHANK proteins do not directly bind to glutamate receptors in the PSD but SHANK1–3 have the capability to link the ionotropic glutamate receptors to the metabotropic glutamate receptors by dimerization and molecular interaction with other scaffold proteins. Compared to DLGAP1–4 and DLG4, SHANK1–3 are longer proteins and have more domains (see Fig. 2b). Close to the N-terminal SHANK1–3 has a SHANK/ProSAP (SPN) domain followed by 7 Ankyrin (Ank) repeats. Like DLG4, SHANKs also have a SH3 domain and one PDZ domain. Downstream of PDZ, a proline rich domain is found, which is followed by a sterile alpha motif, SAM, domain close to the C-terminal. The SPN domain is largely uncharacterized. However, two binding partners have been reported for the Ank repeats, the protein alpha-Fodrin [41] and the Sharpin protein [42]. Both proteins bind to the cytoskeleton [43, 44]. Interestingly, the long isoform of SHANK2 with the Ank repeats, SHANK2E, seems not to be expressed in the PSD but mostly in epithelial cells [45]. SHANK1–3 has one SH3 domain like DLG4. In SHANK1–3, this domain mediates interaction with the PSD scaffold protein Densin-180. Densin-180 can promote dendritic branching. This feature is however negatively regulated when Densin-180 interacts with SHANK proteins [46]. Close to the SH3 domain, SHANK1–3 have a PDZ domain. The C-terminal of DLGAP1–4 interacts directly with SHANK1–3 via this domain [2] (see Fig. 2a & b). Hereby SHANK1–3 are linked to the postsynaptic plasma membrane via DLGAP1–4, PSD-95 and NMDA receptors. Downstream close to the N-terminal SHANK1–3 have a SAM domain, which bind other SAM domains for multimerization of SHANK1–3 proteins [47]. Multimerization of SHANK1–3 proteins can generate a network in the PSD that link numerous proteins to the postsynaptic receptors. This network also enables downstream signalling cascades from the postsynaptic glutamate receptors in which DLGAP proteins are an important element. Finally, SHANK1–3 also connects to the Homer family, via the proline rich domain [20].

##### The Homer family

The Homer family consists of 3 proteins (Homer1–3) with various isoforms encoded by 3 *HOMER* genes. The Homer proteins have a function in neuronal development [48] and they play a central role in the PSD because these proteins bind to group I mGluRs and regulate the activity hereof [49, 50]. *Homer1* has been linked to mental retardation syndromes in Fragile X mental retardation patients and in *Fragile mental retardation 1 (FMR1)* knockout mice [51, 52]. Abnormal spine densities were found in the *FMR1* knockout mice, which support the importance for Homer proteins in neuronal development and proper PSD function.

Homer1–3 have two known domains. The first domain is the highly conserved Ena/VASP Homology 1 (EVH1) domain and the second domain is a coiled coil (CC) domain (see Fig. 2b). The EVH1 domain is located near the N-terminal and with this domain Homer1–3 interacts with the proline rich domain of SHANK1–3 [20]. In addition Homer1–3 have the ability to interact with group I mGluRs that also have a proline rich domain [21]. DLGAP1–4 is not directly associated with Homer1–3. However, Homer1–3 are interesting because they connect DLGAP1–4 to group I mGluR via SHANK1–3 (see Fig. 2b). Like DLG4 and SHANK1–3, Homer1–3 also have the capability to dimerize. The CC domain can bind other CC domains for homodimerization [53].

##### Stargazin

DLGAP proteins indirectly interact with Stargazin via DLG4, which is interesting because it couples DLGAP proteins to the AMPA receptor. Stargazin is a tetraspanning transmembrane protein also known as calcium channel, voltage-dependent, gamma subunit 2. Stargazin shows structural resemblance to calcium channel, voltage-dependent, gamma subunit 1, which is a subunit in voltage gated calcium channels. Thus, it was first believed to be a calcium channel subunit [54], Stargazin is not a genuine subunit of calcium channels [55]. Stargazin is well known from the stargazer mouse, a model of ataxia and epilepsy where the *Stargazin* gene is deleted [56, 57]. Stargazin was found to interact with AMPA receptors and regulate their synaptic targeting, surface trafficking and trafficking to endosomes in ion channel scaling. This regulation require the C-terminal of Stargazin that interacts with the PDZ domain of DLG4 [3, 58–60]. Stargazin also plays an important role in the function of AMPA receptors. Stargazin can modulate the biophysical properties and channel gating of AMPA receptors [61–64]. Moreover, Stargazin can increase the efficacy of agonists on AMPA receptors and it can also act to slow down deactivation of AMPA receptors after glutamate binding [62, 65, 66].

### DLGAP1–4 expression in the brain

**Fig. 3:**
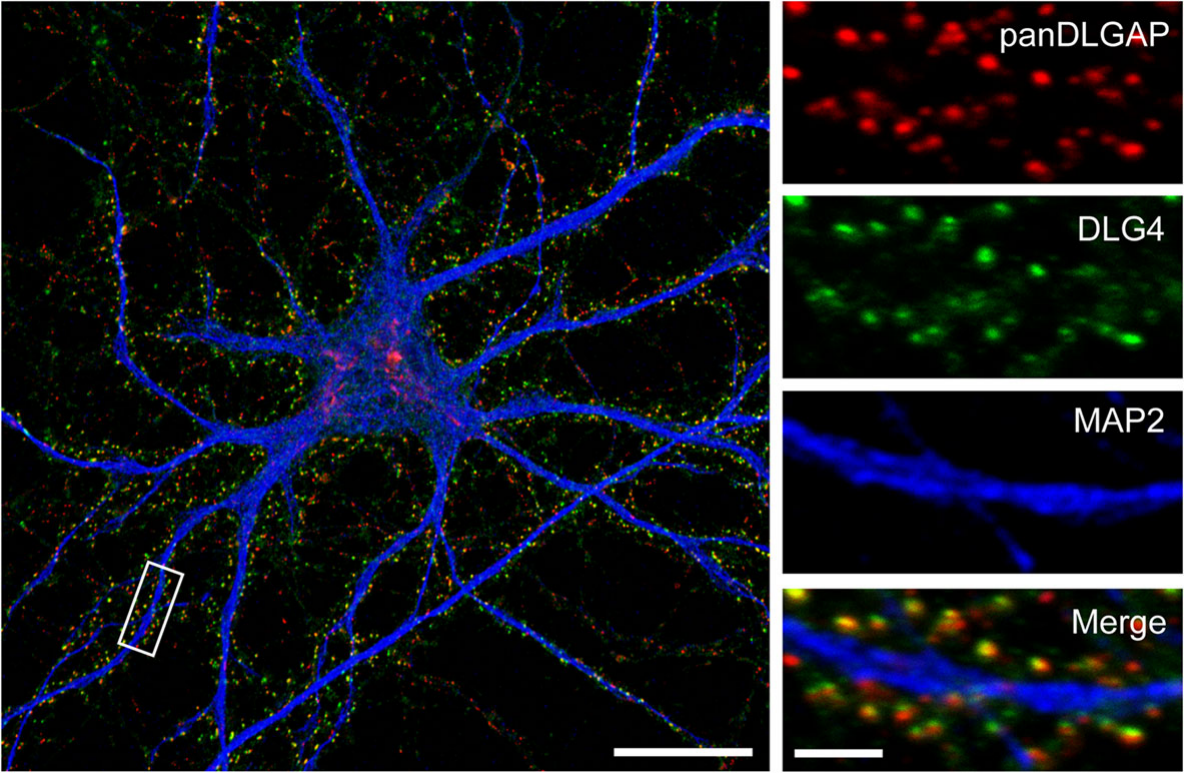
DLGAPs are proteins of the postsynaptic density. The postsynaptic localization of DLGAPs in a cultured rat hippocampal neuron as revealed by a panDLGAP immunostaining. The excitatory postsynapses were visualized with an antibody directed against PSD-95 and MAP-2 used as a dendritic marker. Left scale bar, 20 μm; right scale bar, 3 μm

DLGAP1–4 are expressed throughout the body but in general they are expressed in much higher levels in the brain [6]. DLGAP1–4 are mainly localized in the dendrites and the postsynapse of excitatory synapses (see Fig. 3) [18, 67, 68]. In the brain the expression of DLGAPs is widespread and the different isoforms show a somewhat different expression pattern. In situ hybridization experiments on rat and mouse brain tissue show that *Dlgap1* mRNA is expressed in cortex, hippocampus, olfactory bulb, striatum, thalamus as well as in both granule layer and Purkinje cells of cerebellum (see Table 2) [6, 68]. The expression of *Dlgap2* resembles that of *Dlgap1* with expression in cortex, hippocampus and olfactory bulb. However, *Dlgap2* is highly expressed in the striatum and interestingly *Dlgap2* is the only *Dlgap* that is not expressed in the cerebellum and in the thalamus [68]. Expression of *Dlgap3* mRNA is observed throughout the mouse brain including the cortex, thalamus, retina and olfactory bulb with high levels in the hippocampus, striatum and granule cells of cerebellum [68]. In cortex and cerebellum, the expression level of DLGAP3 varies in the postnatal rat brain. Around 3 weeks after birth the expression level peaks [69]. There are three transcript variants expressed from the *DLGAP4* locus, which are all present in the brain. Transcript variant 1 however, is totally brain specific whereas transcript variant 2 and 3 show tissue-wide expression [14]. In the brain *DLGAP4* shows high expression in the hippocampus, striatum, thalamus, amygdala, substantia nigra and in the Purkinje cells of cerebellum [14, 18, 68, 69].

**Table 2:** The table shows where the DLGAP subtypes are expressed in the brain (Brain region, second column)

DLGAP subtype	Brain region	Brain region	References
DLGAP1	Cortex	↑	[6, 68]
	Hippocampus	↑	
	Cerebellum	↑	
	Olfactory bulbs	↑	
	Striatum	↓	
	Thalamus	M	
DLGAP2	Cortex	M	[68]
	Hippocampus	↑	
	Olfactory bulbs	M	
	Striatum	↑	
DLGAP3	Cortex	M	[68]
	Thalamus	M	
	Retina	M	
	Olfactory bulbs	M	
	Hippocampus	↑	
	Striatum	↑	
	Cerebellum	↑	
DLGAP4	Hippocampus	↑	[14, 18, 69]
	Striatum	↑	
	Cerebellum	↑	
	Thalamus	M	
	Amygdala	M	
	Substantia nigra	↓	

In the third column the expression level for the specific brain region is indicated. ↑; indicates high expression, M; indicates medium expression and ↓; indicates low expression levels. In the fourth column relevant references are linked

### Functional role of DLGAP proteins

#### DLGAP proteins are involved in ion channel scaling at the synapse

The DLGAP1 protein is enriched in the synapse where it acts as a scaffold protein and contributes to synaptic scaling [8]. Synaptic scaling, a form of homeostatic synaptic plasticity, is a function of the excitatory synapses to reset the neuronal firing rate to “normal” levels. The firing rate is normalized by a change in the postsynaptic response in every synapse of a neuron. The change in postsynaptic response results from alterations in the activity of neurotransmitter receptors like AMPA and NMDA receptors. For example, NMDA receptor scaling is regulated via the number of NMDA receptors at the synapse. The number of NMDA receptors functions as a bidirectional feedback mechanism. When the synapse is inactive, NMDA receptors are transported from the endoplasmic reticulum to the synapse. When the synapse is active, synaptic NMDA receptors undergo endocytosis and export of NMDA receptors from the endoplasmic reticulum slows down [70].

**Fig. 4:**
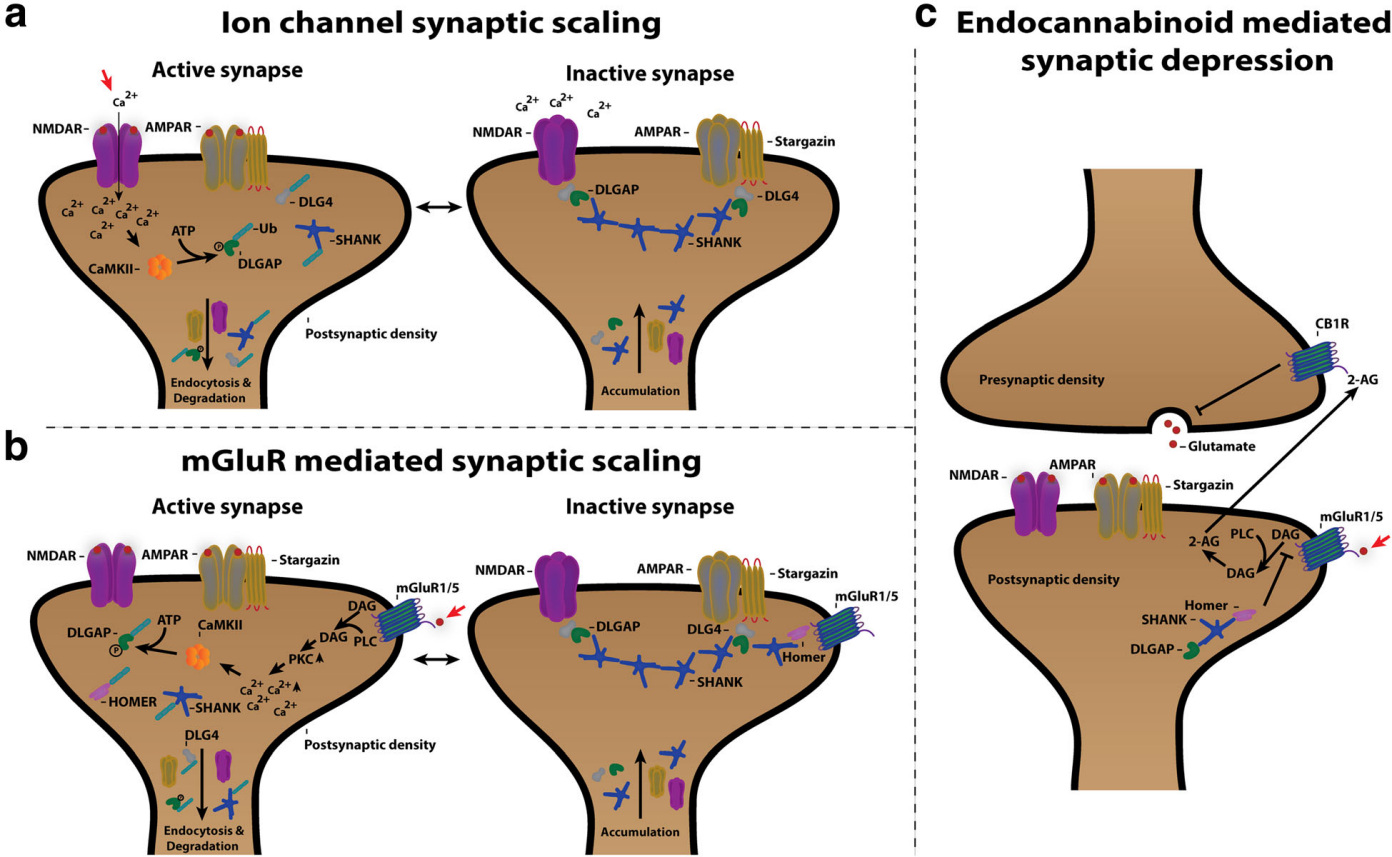
**a** Proposed model of the states in ion channel mediated synaptic scaling. In the active synapse calcium influx through the NMDA receptor activates CaMKII that utilizes ATP to phosphorylate DLGAP that causes dissociation from DLG4 and SHANK and prones them for ubiquitination (Ub). Subsequently, DLGAP, DLG4 and SHANK are degraded, which results in endocytosis of the AMPA and NMDA receptors. During synaptic inactivation the scaffold proteins DLGAP, DLG4 and SHANK accumulate together with the AMPA and NMDA receptors that are incorporated in the membrane. **b** Proposed model of mGluR mediated synaptic scaling displayed as an equilibrium between the active and the inactive synapse. The synaptic scaling is initiated by activation of mGluR1/5 by glutamate followed by PLC-mediated membrane release of DAG. The release of DAG leads to activation of PKC and release of intracellular Ca^2+^. The increase in calcium ions activates CaMKII that phosphorylates DLGAP and Homer. After phosphorylation DLGAP dissociates from DLG4 and SHANK, and Homer dissociates from mGluR1/5 and SHANK. DLGAP, DLG4, Homer and SHANK are then ubiquitinated (Ub) and degraded. With no intracellular bound scaffold proteins, NMDA receptors and AMPA receptors are internalised and removed from the synapse. In the inactive synapse after activation the scaffold proteins DLGAP, DLG4, Homer and SHANK accumulate together with AMPA receptors and NMDA receptors that are incorporated into the membrane. **c** Working model of endocannabinoid-mediated synaptic depression that is initiated by glutamate stimulation of mGluR1/5 that leads to PLC-mediated release of DAG. Then, DAG is converted to the endocannabinoid 2-AG that is transported to the synaptic cleft where it binds to the endocannabinoid receptor CB1R. After activation of CB1R, glutamate release from the presynapse to the synaptic cleft is halted, which leads to the depression. Presence of DLGAP in the PSD inhibits this endocannabinoid synaptic depression most likely via a DLGAP-SHANK-Homer- mGluR1/5

Synaptic scaling and the turnover of NMDA receptors are supplemented by a turnover of proteins in the PSD. The level of the scaffold protein DLG4, which interacts with the cytoplasmic tail of NMDA receptors (reviewed here: Sheng [71]) is reduced during NMDA receptor activity. Reduction of DLG4 at synaptic sites is a result of ubiquitination and proteasomal degradation [72, 73]. DLGAP proteins appear to control the DLG4 degradation indirectly. Upon dissociation of the DLGAP 14-a.a. domain from the DLG4 GK domain, both DLGAP1 and DLG4 are ubiquitinated and degraded [8, 74]. The DLG4-DLGAP1 interaction is disrupted by phosphorylation of DLGAP1, which is catalyzed by the Ca^2+^/calmodulin-dependent protein kinase II alpha chain (αCaMKII). αCaMKII is activated by influx of Ca^2+^ ions through the NMDA receptors upon activation. The scaffold protein SHANK is also downregulated at the synapse as a result of DLGAP1 phosphorylation [8]. The phosphorylation most likely disrupts the interaction of the C-terminal of DLGAP1 with the PDZ domain of SHANK (see Fig. 4a) [75].

During synaptic inactivation DLGAP1, DLG4 and SHANK are, like the NMDA receptors, accumulating at the synapse again. DLGAP1 seems to be the linker in this accumulation. However, DLGAP1, DLG4 and SHANK are equally dependent on each other for accumulation (see Fig. 4a) [76]. It is believed that the scaffold proteins and NMDA receptors have a fast turnover with a short lifespan at the synapse [77].

DLGAP1 turnover seems to be important in synaptic scaling mediated by influx of Ca^2+^ ions through the NMDA receptors but it also plays a role in AMPA receptor scaling. It is known that DLG4 indirectly binds to AMPA receptors through Stargazin [3]. Further, DLGAP proteins interact with DLG4 as described above and phosphorylation of DLG4 by protein kinase A (PKA) leads to removal of AMPA receptors from the membrane by endocytosis [72]. Research has also shown, that loss of DLGAP3 causes silencing of AMPA receptors at the postsynapse [78]. In addition, overexpression of mutant DLGAP1 in hippocampal neurons eliminate homeostatic regulation of AMPA receptor surface expression [8].

DLGAP proteins clearly have a central role in ion channel synaptic scaling including both NMDARs and AMPARs. This function of DLGAPs appears to be achieved in concert with DLG4, SHANK, αCaMKII and possibly Stargazin.

#### DLGAP proteins control metabotropic glutamate receptor group I induced synaptic scaling

The group I metabotropic glutamate receptors (mGluRs) belong to a class of receptors in the mGluR family that is involved in synaptic scaling [79]. mGluRs are G-protein coupled receptors (GPCR) that bind glutamate. Upon glutamate binding of group I mGluRs, phospholipase C (PLC) hydrolyses phospholipids in the membrane. Subsequently inositol 1,4,5-triphosphate (IP3) and diacyl glycerol (DAG) are released, which can lead to activation of protein kinase C (PKC) and increased intracellular levels of Ca^2+^ (see Fig. 4b). The group I mGluR family includes two receptors, namely mGluR1 and mGluR5. Group I mGluRs can modulate NMDA and AMPA receptor activity and induce synaptic scaling upon activation. The scaffold protein Homer seems to be required for group I mGluR induced synaptic scaling [79] and for the crosstalk between group I mGluRs and the NMDA and AMPA receptors [80]. The crosstalk is likely mediated through a DLG4-DLGAP-SHANK-Homer complex [21]. Homer binds to the C-terminal of group I mGluR [81] and the coupling of Homer to the group I mGluR and other interaction partners in the PSD is, like for DLGAP1, also controlled by phosphorylation catalysed by CaMKII [82]. In addition to Homer, DLGAPs are also believed to play an important role in group I mGluR induced synaptic scaling. This is due to the essential binding to both DLG4 and SHANK, and research show that loss of DLGAP2 causes significant downregulation of Homer and AMPA receptors [83]. DLGAP’s role in group I mGluR synaptic scaling is probably not much different from its role in ion channel scaling. DLGAPs are likely phosphorylated by CaMKII upon group I mGluR activation. Phosphorylation of DLGAP and Homer leads to degradation or removal of DLGAP, Homer, DLG4 and SHANK from the PSD. Upon degradation or removal of scaffold proteins, the NMDA and AMPA receptors are unstable and subsequently removed from the membrane via endocytosis (see Fig. 4b).

#### The role of endocannabinoids in synaptic depression is guarded by DLGAP

The group I mGluRs can regulate synaptic plasticity via release of intracellular Ca^2+^ as described above. Another way group I mGluRs can regulate synaptic plasticity is through endocannabinoid mediated synaptic depression. Endocannabinoids are a class of lipids that are synthesized from membrane-bound phospholipids. The synthesis occurs after activation of group I mGluRs where the phospholipids are converted into endocannabinoids via different cleaving mechanisms. For example, DAG that is released from the membrane by PLC after group I mGluR activation is converted to the endocannabinoid 2-Arachidonoyl glycerol (2-AG). After synthesis the endocannabinoids are transported into the synaptic cleft and to the presynapse where the endocannabinoid receptor 1 (CB1R) is located. The CB1R is a GPCR and upon endocannabinoid binding, downstream signalling is initiated. One downstream signalling cascade results in inhibition of neurotransmitter release from the presynapse (see Fig. 4c) [84]. The scaffold protein Homer1 that binds group I mGluRs seems to play a role in the endocannabinoid signalling [85]. Homer1 expression regulates endocannabonoid production as a result of mGluR stimulation. Besides Homer1, DLGAP3 also regulates endocannabinoid mediated synaptic depression. In *Dlgap3* knockout mice, endocannabinoid-dependent synaptic depression is increased in the striatum in a mGluR dependent manner [7]. This indicates that the presence of DLGAP proteins in the PSD activates or promotes synaptic transmission by inhibiting mGluR-mediated endocannabinoid signalling. The scaffold protein SHANK binds both DLGAP and Homer why DLGAP3 most likely controls endocannabinoid signalling through a DLGAP3-SHANK-Homer-group I mGluR complex (see Fig. 4c).

### DLGAP proteins in neurological and psychiatric disorders

The DLGAP proteins are expressed in the postsynapse and interact with several proteins involved in the function and maintenance of the NMDA, AMPA and group I mGluR glutamate receptors. DLGAPs play an important role in the PSD and even small changes in expression of DLGAPs could have severe consequences in the signalling within the PSD. It is therefore not surprising that all DLGAPs have been linked to various psychiatric and neurological disorders including schizophrenia, OCD and cerebellar ataxia (see Table 1) [9, 11, 14].

#### Schizophrenia and DLGAPs

Schizophrenia is a widespread, complex mental disorder that is characterized by symptoms like delusions, hallucinations and abnormal social behaviour. It affects approximately 1% of the population worldwide [86]. Schizophrenia has a high degree of heritability however, a large fraction of patients do not have a family history which can be explained by sporadic mutations [87, 88].

Schizophrenia is functionally associated with the NMDA receptors [89, 90]. One hypothesis behind the NMDA receptor influence in schizophrenia is explained by altered glutamate signalling as a result of reduced or increased incorporation of NMDA receptors in the postsynaptic membrane [91, 92]. As explained in section 6, DLGAPs influence NMDA receptor synaptic scaling via DLG4 and both DLG4 and DLGAP1 have been linked to schizophrenia. The DLG family members, DLG1 and DLG2, which also interact with DLGAPs and NMDA receptors [93–95] have been linked to schizophrenia as well [35–37].

Analysis of brain samples from post mortem schizophrenic patients revealed that the level of DLG4 was downregulated in anterior cingulate cortex [33]. Another study demonstrated increased expression of DLGAP1 in the nucleus accumbens [9]. Moreover, the expression level of DLG1 and DLG2 was also found to be significantly skewed in a schizophrenia rat model and in schizophrenic patients [32, 96–98]. The deregulation of DLGs and DLGAP1 in these patients could be an indication of malfunction of NMDA receptors. Especially the NMDA subunit, GluN2A shows altered expression in schizophrenic patients [91, 92]. The schizophrenia-related upregulation of DLGAP1 expression could be the result of a feedback mechanism where the neurons are trying to re-establish the normal function and signalling of the GluN2A containing NMDA receptor.

After the discovery of the potential role of DLGAP1 in schizophrenia, related gene family members have also been analysed extensively in genetic studies. Multiple single nucleotide variations (SNVs) have been reported in *DLGAP2* and *DLGAP3* genes in schizophrenia patient cohorts [99, 100]. In *DLGAP3* two SNVs were identified in the C-terminal, one of which was located in a domain which is believed to affect the post-translational modification of the DLGAP3 protein. It was speculated that all the *DLGAP3* SNVs would impact the interaction with protein kinases and thereby the function of DLGAP3 [99]. No functional studies have yet been conducted on *DLGAP2* and *DLGAP3* in relation to schizophrenia. *DLGAP2* and *DLGAP3* have only been linked to schizophrenia in genetic screens. Yet, genetic changes in *DLGAP2* and *DLGAP3* are considered as susceptibility factors for schizophrenia [99, 100].

#### Other brain disorders and DLGAPs

The DLGAPs have been linked to a number of brain disorders in addition to schizophrenia. *DLGAP2* was identified as a candidate gene for ASD where patients display impaired social interaction and communication skills in addition to having stereotyped interests and behaviours [10, 101, 102].

Animal studies have pinpointed a link between *DLGAP2* and post-traumatic stress disorder (PTSD), which is observed as a result of a traumatic experience. In a rat model of PTSD, a change in methylation and in expression of *Dlgap2* was shown in the hippocampus [103]. In individuals with PTSD the hippocampus is known to be smaller and less activated [104–106]. Moreover, *Dlgap2* knockout (*Dlgap2* ^*−/−*^) mice showed deficits in learning, abnormal social behaviour and intense aggressive behaviour, which is characteristic for both ASD and PTSD [83].

Biochemical studies revealed that specific subunits in the AMPA and NMDA receptors, the scaffold protein Homer1 and αCaMKII were significantly downregulated in the *Dlgap2* ^*−/−*^ mice resulting in disruption of both synaptic transmission and synaptic structure [83].

*DLGAP3* has been associated with OCD, which arises from anomalous signalling in the striatum and develops into a phenotype where the patients need to check or perform certain things repeatedly. *Dlgap3* knockout (*Dlgap3* ^−/−^) mice showed increased self-grooming bouts compared to wild type mice, which resulted in lesions in their head and neck. In addition, the *Dlgap3* ^−/−^ mice exhibited increased anxiety-like behaviour, which is also characteristic for OCD [11].

Since *Dlgap3* ^−/−^ mice showed OCD-like symptoms, patients with the grooming disorder, trichotillomania (hair-pulling disorder) and OCD were genotyped for SNVs in the *DLGAP3* locus [12]. An association was found between variations in the *DLGAP3* gene and trichotillomania, which appeared to be familial. However, a coherence between variants in the *DLGAP3* gene and OCD was not obvious [12]. Other studies have nonetheless linked genetic variants in *DLGAP3* to OCD-like symptoms. In patients with OCD and trichotillomania, multiple and rare missense mutations were found in the *DLGAP3* gene [107].

The fourth *DLGAP* gene, *DLGAP4,* has been related to cerebellar ataxia. Our group had discovered a familial translocation between chromosome 8 and 20 t(8:20) segregating with early-onset cerebellar ataxia [108]. This translocation resulted in symptoms including ataxia, clumsiness, impaired hand coordination and tremors. The translocation disrupted the *DLGAP4* locus and separated the promoter and the first exon from the rest in transcript variant 1, which is brain-specific. In addition, the translocation disrupted the *DLGAP4* promoter associated CpG island which lead to epigenetic changes and increased expression of the *DLGAP4* transcript variant 2 [14].

The misregulation of *DLGAP4* could very likely lead to altered surface expression of both ionoptropic and metabotropic glutamate receptors in the PSD. Deregulation of glutamate receptor turnover, could potentially be followed by a faulted glutamate signalling from the presynapse to the postsynapse leading to the reported phenotype. In fact, both loss of AMPA receptors and loss of group I mGluR signalling have previously been associated with cerebellar ataxia [109–111].

## Conclusions

In this review we have focused on members 1 to 4 of the DLGAP protein family with regard to their function in the brain and involvement in neurological diseases. The DLGAP proteins have multiple domains and act as scaffold proteins in the PSD where they enable crosstalk between metabotropic and ionotropic glutamate receptors via other scaffold proteins including DLG4, SHANK and Homer [1–5, 21, 47, 49, 50, 58–60]. DLGAPs are believed to control synaptic scaling as a result of NMDA receptor, AMPA receptor and group I mGluR activation [3, 5, 7, 8, 58–60]. Undoubtedly further research on this topic would be of great value to decipher the role of the individual DLGAP proteins and the interplay between the DLGAP proteins in neuronal synapses. To understand the implication of DLGAPs in neurological disorders will be of great importance. Multiple studies have been conducted on DLGAPs in relation to schizophrenia [37, 86, 112–114]. Specifically, there is heavy literature on involvement of DLGAP1 in schizophrenia and suggesting DLGAP2 as a candidate gene for ASD [10, 100, 101] and PTSD [83, 102]. It is likely that mutations in *DLGAP2* could lead to the symptoms seen in ASD and PTSD patients. The data convincingly depicts the *DLGAP3* gene as a disease gene for both OCD and trichotillomania [11–13, 106]. In contrast to DLGAP1–3, the general literature on DLGAP4 is very limited. *DLGAP4* is proposed as a candidate gene for cerebellar ataxia [14] but *DLGAP4* was never investigated in studies of the Stargazer mouse, a mouse model of cerebellar ataxia [3, 54, 56]. More research and data is expected in the coming years to clarify the role of DLGAP1, 2, 3 and 4 in synaptic plasticity and their involvement in schizophrenia and neurological diseases.

## Abbreviations

AMPA: α-amino-3-hydroxy-5-methyl-4-isoxazolepropionic acid
Ank: Ankyrin
ASD: Autism spectrum disease
ASD: Autism spectrum disorder
CB1R: Endocannabinoid receptor 1
CC: Coiled coil
DAG: Diacyl glycerol
DLC: Dynein light chain
DLGAP1-4: Discs large associated protein 1, 2, 3 and 4
DLGAPs: Discs large associated proteins
DLGs: Discs large scaffold proteins
EVH1: Ena/VASP Homology 1
FMR1: Fragile mental retardation 1
GH1: GKAP homology
GK: Guanylate kinase
GKAP: Guanylate kinase associated protein
GPCR: G-protein coupled receptors
Homer1-3: Homer family consisting of 3 proteins
IP3: Inositol 1,4,5-triphosphate
MAGUKs: Membrane-associated guanylate kinases
mGluRs: Metabotropic glutamate receptors
NMDA: N-methyl-D-aspartate
OCD: Obsessive compulsive disorder
PDZ: Zonula occludens-1 protein
PKA: Protein kinase A
PKC: Protein kinase C
PLC: Phospholipase C
PSD: Postsynaptic density
PTSD: Post-traumatic stress disorder
SAPAP1-4: SAP90/PSD95-associated protein 1–4
SH3: Src Homology 3
SHANK: Src-homology (SH3) and multiple ankyrin repeat domain proteins
SHANK1-3: SHANK proteins
SNV: Single nucleotide variation
SPN: SHANK/ProSAP
